# Accelerate the Highly Efficient Development of mRNA Vaccines Through Advanced Computational Methods

**DOI:** 10.1002/mco2.70612

**Published:** 2026-02-08

**Authors:** Ruichu Gu, Duanmiao Si, Dingwei Lei, Xiaoxue Xie, Yongge Li, Han Wen

**Affiliations:** ^1^ DP Technology Beijing China; ^2^ Beijing Advanced Center of RNA Biology (BEACON) Peking University Beijing China; ^3^ School of Pharmaceutical Sciences, Peking University Beijing China; ^4^ School of Life Sciences, Peking University Beijing China; ^5^ AI for Science Institute Beijing China; ^6^ Institute for Advanced Algorithms Research Shanghai China

**Keywords:** artificial intelligence, computational methods, delivery system, modification, mRNA vaccine, sequence optimization

## Abstract

mRNA medicine is an emerging therapeutic approach that utilizes messenger RNA to synthesize functional proteins directly within target cells. This technology offers notable advantages including rapid development cycles, diverse therapeutic applications, and adaptable platform design for various diseases. However, mRNA therapeutic development faces substantial challenges, particularly in determining optimal mRNA sequences and developing effective delivery systems that ensure stability and achieve precise delivery. Current development processes often involve extensive experimental screening, highlighting the need for more efficient computational approaches. This review first introduces fundamental concepts in the mRNA vaccine field and systematically analyzes the roles and limitations of computational tools in advancing mRNA vaccine development across three key areas: sequence optimization, modification strategies, and delivery system optimization. Finally, we present the current application status of mRNA vaccines and discuss future prospects, highlighting emerging computational opportunities that may shape next‐generation mRNA vaccine development. This review spans the entire mRNA vaccine development pipeline, providing a foundational resource for researchers and facilitating technological advancement in this rapidly evolving field.

## Graphical abstract

This review explores the recent advancements in applying computational methods to optimize mRNA vaccines, with a primary focus on improvements in sequence design and delivery systems.

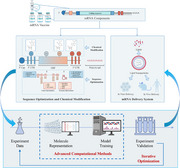

## 1 Introduction

**FIGURE 1:**
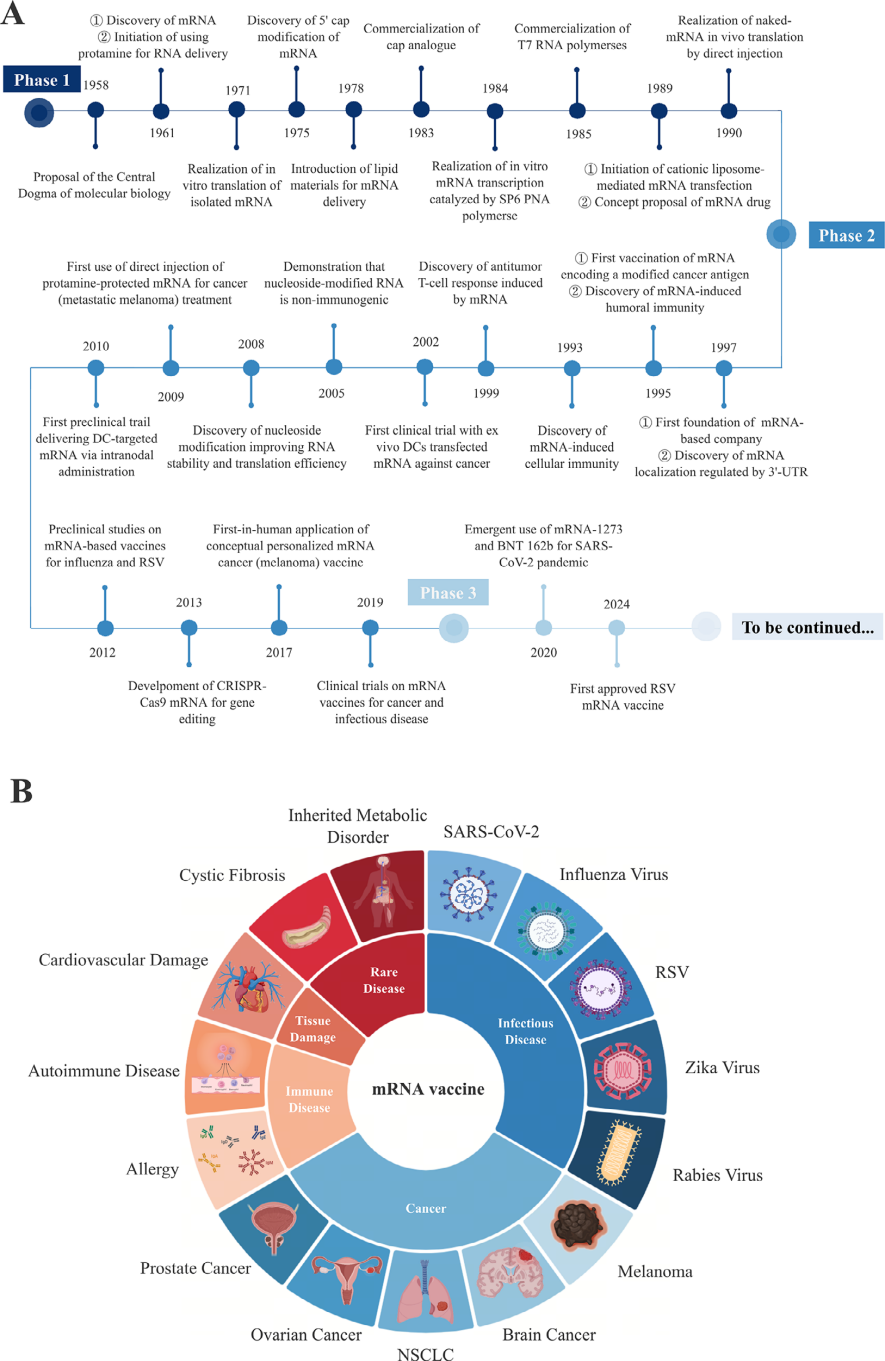
Development and applications of mRNA‐based vaccines. (A) The development of mRNA‐based vaccine: The development of mRNA vaccine can be divided into three phases: Stage 1 (1961–1990), mRNA discovery, in vitro transcription, chemical modification, and delivery system exploration. Stage 2 (1990–2019), trials of mRNA vaccines in different fields of application. Stage 3 (2019 to present), the successful application of mRNA vaccine in SARS‐CoV‐2 and the expansion of other indications [3, 16]; and (B) the applications of mRNA‐based vaccine: mRNA vaccines are now being explored and tested in a variety of applications, including infectious diseases, cancers, immune diseases, tissue damage, and rare diseases [6]. DC, dendritic cell; RSV, respiratory syncytial virus; UTR, untranslated region. (Created with BioRender.com)

Messenger RNA (mRNA) is a single‐stranded ribonucleic acid molecule that serves as an intermediary carrier of genetic information from DNA to protein synthesis. Since its discovery in 1961, mRNA technology has evolved from a fundamental biological concept to a revolutionary therapeutic platform. The rapid development and approval of mRNA vaccines by Pfizer/BioNTech and Moderna during the SARS‐CoV‐2 pandemic marked a pivotal moment in vaccine development [1, 2], demonstrating the remarkable potential of mRNA‐based therapeutics and establishing their clinical viability across various medical applications [3]. Since the discovery of mRNA in 1961, research into its structure, function, and related technologies has advanced rapidly, culminating in achievements such as the positive phase II results of BNT111 reported on July 30, 2024 (Figure 1A).

While mRNA therapy demonstrates remarkable potential for disease prevention and treatment, technical challenges associated with mRNA stability and immunogenicity control continue to impede therapeutic development and clinical advancement. The primary obstacles revolve around molecular architecture optimization and in vivo delivery methodologies [4, 5, 6, 7], including mRNA stability, immunogenicity regulation, and efficient cellular uptake [8, 9]. Concurrently, the stability and translation efficiency of the vaccine can be further improved by sequence optimization and suitable chemical modification [10, 11, 12]. However, traditional experimental approaches for addressing these challenges are inherently time‐consuming, costly, and limited by the complexity of biological systems and the extensive parameter space of mRNA design variables [12, 13]. These limitations have created an urgent need for more efficient, systematic approaches to mRNA vaccine optimization.

Recent advances in computational methods have created unprecedented opportunities to process large‐scale experimental data and tackle previously intractable biological problems [14]. These computational approaches demonstrate significant potential for enhancing multiple aspects of mRNA vaccine design, including sequence optimization, chemical modifications, and delivery system development, thereby improving both efficacy and safety in clinical applications [15]. Artificial intelligence and deep learning (DL) technologies, in particular, offer powerful capabilities for navigating the complex landscape of mRNA vaccine optimization and predicting optimal design parameters.

However, current applications of computational tools in mRNA vaccine development remain fragmented across disparate research domains, lacking systematic integration and comprehensive analysis. This fragmentation limits the full realization of computational potential and hinders the establishment of standardized optimization workflows. Consequently, there is an urgent need for a comprehensive review to consolidate existing computational advances, evaluate their effectiveness, and identify strategic directions for future algorithmic development in mRNA vaccine design.

This review provides a comprehensive examination of computational approaches in mRNA vaccine development, systematically analyzing three core applications in sequence optimization, chemical modifications, and delivery system design while critically evaluating current methodological limitations and identifying emerging opportunities for algorithmic advancement. The review is structured to first establish fundamental mRNA vaccine concepts, then systematically analyze computational applications across the three core domains, assess current limitations and applications, and conclude with future prospects for algorithmic advancement. This framework provides researchers with a strategic roadmap for integrating computational strategies into mRNA therapeutic design and advancing future vaccine development.

## 2 Overview of mRNA Vaccine Technology

This section provides a comprehensive overview of mRNA vaccine, covering three aspects: the primary components of mRNA and their respective functions, the underlying immunological mechanisms by which mRNA vaccines elicit protective immune responses, and the current developmental status of mRNA vaccine.

### 2.1 Components of mRNA Vaccines

**FIGURE 2:**
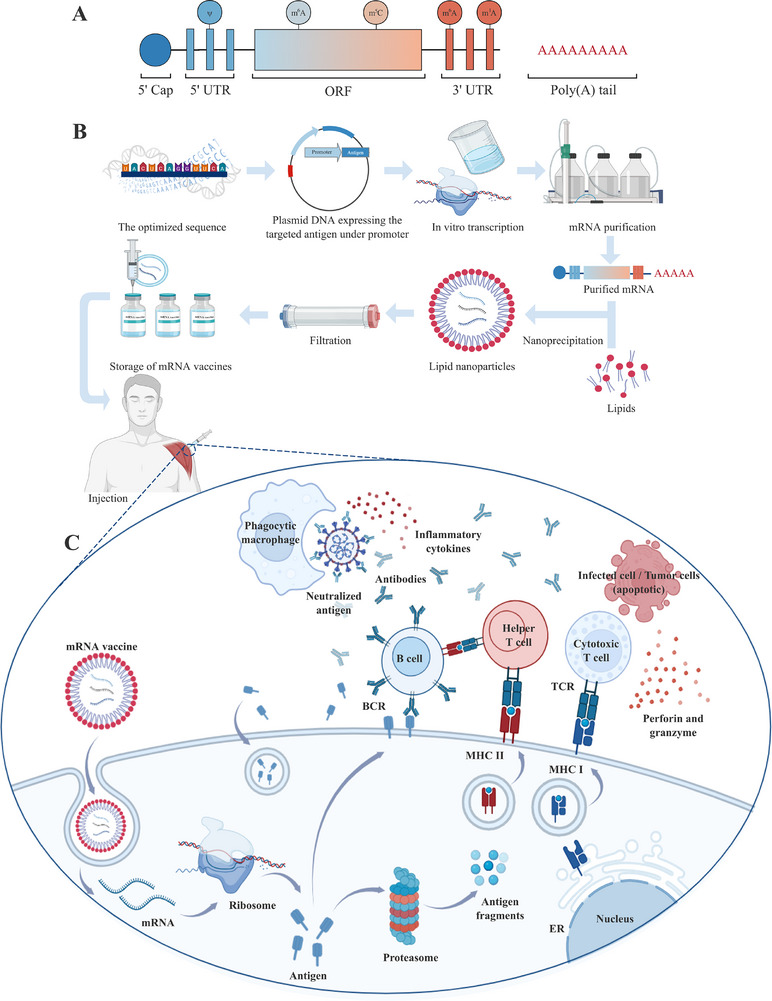
Structural elements, development process, and mechanism of mRNA vaccines. (A) The structural elements of mRNA: mRNA contains five structural elements, including the 5′ cap, the 5′ UTR, the ORF, the 3′ UTR, and the 3′ poly(A) tail; and (B) the development process of mRNA vaccine: sequence design and optimization, plasmid DNA expression, in vitro transcription, mRNA purification, formulation with lipid nanoparticles, filtration, and storage. (C) The mechanism of mRNA vaccine: mRNA delivery and expression, antigen processing and presentation, adaptive immune activation, and innate immune synergy. BCR, B cell receptor; ER, endoplasmic reticulum; MHC, major histocompatibility complex; ORF, open reading frame; TCR, T cell receptor; UTR, untranslated region. (Created with BioRender.com)

The fundamental component of an mRNA vaccine is the in vitro transcribed (IVT) mRNA molecule, which directly controls the production of proteins inside the cell. The mRNA is composed of five structural components, including the 5′ cap, the 5′ untranslated region (UTR), the open reading frame (ORF), the 3′ UTR, and the 3′ poly(A) tail (Figure 2A).

In eukaryotes, the 5′ cap, which contains a 7‐methylguanosine (m7G) attaching nucleotide through a 5′–5′ triphosphate bridge (ppp) at the 5′ end of the mRNA sequence, has the ability to improve the stability of mRNA and regulate cap‐dependent protein synthesis [16, 17]. UTRs are noncoding segments located at the upstream (5′ UTR) and downstream (3′ UTR) regions of the open reading frame (ORF) and are crucial for controlling mRNA translation and protein expression [3, 16, 18, 19, 20]. Both the 5′ and 3′ UTRs influence mRNA's translation efficiency and stability [21], among which the 5′ UTR primarily participates in the initiation of translation [22], while the 3′ UTR predominantly regulates the stability and decay of mRNA [23]. The 3′ poly(A) tail, typically consisting of 10–250 adenine ribonucleotides, plays a crucial role in determining the translational efficacy and decay of mRNA molecules, with its dynamic length being the key regulatory factor [3, 16, 24]. The ORF represents the protein‐coding region of the mRNA molecule. Adopting appropriate codon optimization strategies in ORF is a promising way to enhance mRNA vaccine stability and translation efficiency, which will be discussed in the following sequence optimization section [16, 25]. Besides, functional peptides, particularly signal sequences, are critical for optimizing antigen localization in mRNA vaccines [26]. These peptides direct encoded antigens to specific cellular locations such as secretory pathways, cell surfaces, or endosomal compartments to enhance immune recognition and improve vaccine efficacy [27].

### 2.2 Mechanism of mRNA Vaccines

mRNA vaccines are designed to stimulate patients’ immune response through the introduction of synthetic mRNA. Upon injection, mRNA vaccines are internalized by antigen‐presenting cells (APCs) through endocytosis. Following their release into the cytosol, ribosomes translate the mRNA into specific antigenic proteins to activate the immune response through multiple ways. Intracellular antigens are processed by the proteasome into smaller peptide fragments. These fragments are subsequently presented on the cell surface via major histocompatibility complex (MHC) class I molecules for the recognition by cytotoxic T cells. Once activated, these cytotoxic T cells kill infected cells or tumor cells by secreting cytolytic molecules, including perforin and granzyme. Moreover, secreted antigens are recognized by cognate B cells, triggering the production of potent neutralizing antibodies with robust germinal center reactions. Additionally, these antigens can be internalized by cells, where they are degraded in endosomes and presented to helper T cells via MHC class II molecules. Activated helper T cells also play a critical role in the clearance of circulating antigens by stimulating B cells to produce neutralizing antibodies and activating phagocytic cells, such as macrophages, through the release of inflammatory cytokines. This leads to the safe and specific destruction of infected cells or tumor cells and the suppression of disease progression [6] (Figure 2C).

### 2.3 Current Status of mRNA Vaccines

The systematic investigations of mRNA structure and function have greatly improved the efficiency of the mRNA vaccine development process. This process begins with sequencing the genome of the antigen, followed by the design of a target antigen sequence, which is then inserted into a plasmid DNA construct. This plasmid DNA is subsequently transcribed into mRNA using bacteriophage polymerases in vitro. Then, the mRNA transcripts undergo purification through high‐performance liquid chromatography (HPLC) to eliminate contaminants and residual reactants. Once purified, the mRNA is encapsulated with lipids in a microfluidic mixer, facilitating the rapid formation of lipid nanoparticles (LNPs). These nanoparticles are then subjected to dialysis or filtration to remove nonaqueous solvents and any unencapsulated mRNA. Finally, the resulting vaccine solution is stored in sterilized vials, ready for further vaccination [6] (Figure 2B). This mature development process has further resulted in the gradual establishment of a complete industry chain that is both feasible and reliable. The success of developing SARS‐CoV‐2 and RSV mRNA vaccines has confirmed this approach [6, 28]. As a result, this effective method significantly increased the industrial scalability, reduced cost, and shortened the research and development time of mRNA vaccines. From a commercial perspective, this efficiency can be ascribed to the particularly efficient in vitro transcription reaction and the sophisticated large‐scale industrial production systems, which have completely transformed the production of mRNA vaccines [28, 29].

Alongside these advancements, the rapid evolution of delivery systems has further expedited the clinical application of mRNA vaccines, resulting in a variety of delivery vectors. Among these, LNPs have shown the most promise due to their success in siRNA therapies and SARS‐CoV‐2 vaccines (e.g., Comirnaty and Spikevax) [30, 31, 32]. Systematic LNP synthesis and optimization strategies have emerged, increasingly incorporating computational methods to accelerate candidate screening.

The mRNA vaccines offer several benefits, including broad applicability, great effectiveness and safety, and cost‐efficiency [28, 33]. mRNA vaccines can theoretically encode any antigens based on disease characteristics, significantly broadening their potential applications [28]. Unlike conventional vaccines that process exogenous proteins, mRNA vaccines synthesize antigens intracellularly, enabling more effective cellular immune stimulation and enhanced cytotoxic responses against specific pathogens. This leads to a more efficient therapeutic approach for both disease prevention and therapeutic intervention [34]. Moreover, unlike DNA vaccines, mRNA vaccines avoid the potential risk of integration into the host genome, offering a higher safety profile [35].

However, mRNA vaccines have not reached their full potential due to challenges with mRNA instability, immunogenicity, and delivery efficiency in vivo [36]. Consequently, there is ongoing research to investigate methods to effectively tackle the difficulties associated with mRNA. Incorporating computational techniques throughout the development process can resolve current challenges more efficiently while reducing time and cost for market introduction. Particularly for SARS‐CoV‐2 mRNA vaccines, computational methods aid in a number of ways, such as the optimization of clinical trial procedures, sequence design, and modification, to increase overall efficacy and speed up vaccine production. This creates a more conducive atmosphere for the advancement of novel mRNA vaccine technologies [37].

## 3 mRNA Sequence Optimization

**FIGURE 3:**
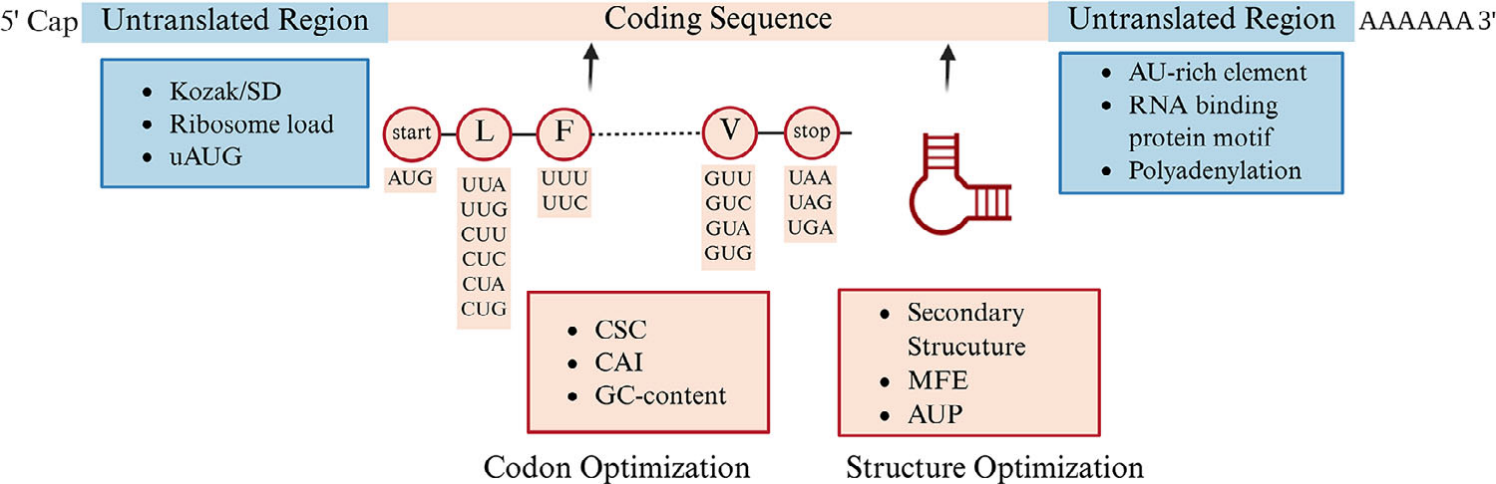
Overview of mRNA sequence design. The general principles of mRNA sequence design are divided into two main sections: the UTRs and the coding sequence (CDS). Key considerations for the 5′ UTR include reducing upstream ORFs (uORFs) and upstream AUGs, minimizing secondary structures, and introducing Kozak/SD sequence to enhance translation initiation. The 3′ UTR focuses on incorporating AU‐rich elements and utilizing natural UTR sequences to enhance mRNA stability and translation efficiency. The CDS section encompasses codon optimization and structure‐based design strategies to ensure efficiency. AUP, average unpaired probability; CAI, Codon Adaptation Index; CSC, Codon Stabilization Coefficient; Kozak, Kozak sequence; MFE, minimum free energy; SD sequence, Shine–Dalgarno sequence; tAI, tRNA Adaptation Index; uAUGs, upstream start codons; UTR, untranslated region. (Created with BioRender.com)

Sequence optimization is fundamental to mRNA vaccine development, requiring precise fine‐tuning to achieve desired therapeutic outcomes. This section presents various optimization strategies, focusing on both coding and UTRs (Figure 3). We examine approaches for enhancing mRNA stability and translation efficiency, and explore how computational methods advance sequence design through systematic refinement of nucleotide composition and structural elements.

### 3.1 Optimization of the Coding Region

#### 3.1.1 Codon Optimization

A major obstacle in the design of therapeutic mRNA sequences is the presence of codon degeneracy. For example, there are approximately 10^632^ potential sequences to encode the 1273 amino acids of SARS‐CoV‐2 spike protein, a number so large that it defies conventional comparison [38, 39]. Different sequences are associated with unique characteristics that affect the efficiency of mRNA translation and the rate of degradation. This sequence variability substantially influences mRNA vaccine effectiveness and stability. Therefore, selecting optimal codons from this vast array presents both critical importance and significant challenges.

In mammals, optimal codons tend to be GC‐rich, with a pronounced preference for cytosine at the wobble position of a codon [40]. GC pairing is more stable than AU pairing; thus, maximizing GC content could result in a more stable secondary structure. Furthermore, GC‐rich sequences inhibit mRNA decay through specific cellular pathways, enhancing protein expression [41]. Uridine‐rich RNA sequences represent viral genetic signatures that pattern recognition receptors (PRRs) readily detect, triggering unwanted innate immune responses [42, 43]. Therefore, the use of uridines in mRNA vaccines should be minimized.

Numerous attempts have been made to optimize codon optimality. Relative synonymous codon usage (RSCU) served as one of the most widely used [44]. RSCU is defined as the ratio of the observed frequency of a synonymous codon to the expected frequency of a given codon. RSCU values can be calculated as follows:

$$\;\mathrm{RSC}U_{ij}=\frac{X_{ij}}{\;\frac{1}{n_{j}}\;\sum_{1}^{n_{j}}X_{ij}},$$

where $X_{ij}$ represents the number of the *j*th codon for the *i*th amino acid, and $n_{j}\;$ is the number of possible conditions for the *i*th amino acids. Then, they can be used to calculate the Codon Adaptation Index (CAI) [45], which indicates the overall optimality score ranging from 0 to 1:

$$\mathrm{CAI}=\frac{\mathrm{CA}I_{\mathrm{obs}}}{\mathrm{CA}I_{\max}}=\frac{{\left(\prod_{k=1}^{L}\mathrm{RSC}U_{k}\right)}^{1/L}}{{\left(\prod_{k=1}^{L}\mathrm{RSC}U_{k\max}\right)}^{1/L}}\;,$$

where $\mathrm{RSC}U_{k}$ represents the observed RSCU of the *k*th codon of the gene, $\mathrm{RSC}U_{k\max}$ is the corresponding maximum RSCU, and *L* is the number of codons in the gene. Similarly, $\mathrm{CA}I_{\mathrm{obs}}$ is the observed CAI of the gene, and $\mathrm{CA}I_{\max}$ is the corresponding maximum value.

Maximizing the CAI is a commonly employed strategy in designing vaccine sequences. For instance, the approved mRNA vaccines, BNT‐162b2 and mRNA‐1273, incorporate mRNA sequences with significantly improved CAI values, exceeding 0.9, compared to the wild‐type antigen sequence [39]. However, optimizing the CAI index alone is insufficient to enhance overall protein expression.

Alternatively, the tRNA Adaptation Index (tAI) is a tRNA‐centric measure of translation efficiency. Due to the challenges in accurately measuring differential tRNA abundance, proxies such as the number of tRNA genes [46] or tRNA levels obtained through tRNA sequencing (tRNA‐seq) [47, 48] are typically utilized. Although these indices are applicable across entire transcripts, they may exhibit local biases. For example, rare codons are enriched in the first 30–50 codons of endogenous transcripts, which could be explained by the “translation ramp hypothesis” [49]. Therefore, it is recommended to decrease the significance of tAI for codon optimization in the vicinity of the CDS.

Moreover, the Codon Stabilization Coefficient (CSC) is derived from the correlation between each codon's frequency in transcripts and experimental data on mRNA half‐life. By calculating the average CSC for each gene, it has been observed that most mRNAs tend to have a higher prevalence of nonoptimal codons [50]. In summary, GC content, CAI, tAI, and CSC are commonly used metrics in codon optimization. By appropriately optimizing these indices, the characteristics of mRNA can be greatly improved.

#### 3.1.2 Structure‐Based Optimization

In the design of therapeutic mRNA, stability is an additional critical aspect. Given the intricate and diverse nature of RNA structures, precise estimation of mRNA secondary structure is challenging yet crucial. Various methodologies have been explored to analyze RNA secondary structures, including models based on thermodynamic parameters, probabilistic generative models, and approaches reliant on DL [51]. These methods facilitate a deeper understanding of mRNA structure, thereby aiding in optimizing mRNA sequences based on their structural characteristics.

Regarding translation, the structural dynamics of CDSs and UTRs play pivotal roles. CDS regions with minimal secondary structures typically facilitate ribosome movement, thereby enhancing translation efficiency. In contrast, specific secondary structures in UTRs serve as the entry point for the ribosome, thus promoting translation initiation [52]. Supporting this, computational predictions and transcriptome‐wide secondary structure probing have revealed that human CDSs are, on average, slightly less structured than their neighboring UTRs [53, 54]. However, conservation analyses in humans and mice suggested that wobble positions within the CDS are under selective pressure to increase base‐pairing interactions [55].

A commonly used metric for mRNA stability is the minimum free energy (MFE), with lower values indicating the formation of more stable secondary structures [56]. Several efficient computational methods have been proposed to systematically calculate MFE to identify the most stable RNA sequences [57]. For instance, the CDSfold program designs coding sequences with the most stable secondary structure, measured by MFE, in cubic time of sequence length [58]. On the other hand, LinearDesign can find the optimal sequence with the minimum MFE in quadratic time relative to the sequence length, using heuristics beam search to find near‐optimal solutions in linear time [13]. The LinearDesign algorithm leveraged lattice parsing from computational linguistics to optimize mRNA stability and codon usage. Using a deterministic finite automaton (DFA) to encode candidate sequences, LinearDesign demonstrated superior performance across multiple metrics [13]. Another metric for mRNA stability is the average unpaired probability (AUP), which represents the average probability of unpaired nucleotides within a sequence. Based on this metric, a stochastic algorithm called RiboTree was developed to optimize RNA sequences for minimal AUP [59]. These tools facilitate sequence design by optimizing structural stability by adjusting MFE or AUP, thereby assisting in developing more stable RNA sequences.

In conclusion, the optimization of the secondary structure of mRNA leads to a significant improvement in its stability and greatly enhances its translation efficiency [13]. The precise design and optimization of mRNA sequences can be achieved by utilizing metrics such as MFE and AUP in conjunction with sophisticated computational tools and algorithms.

### 3.2 UTR Optimization for Improving Protein Expression

Another crucial element in designing mRNA sequences is the UTRs. UTRs from highly expressed genes are commonly used to enhance the translation efficiency of the designed sequences [39]. Further modulation of protein expression can also be achieved by incorporating AU‐rich elements into the 3′ UTR [60]. The presence of upstream start codons (uAUGs) and upstream ORFs (uORFs) in the 5′ UTR can exert a repressive effect by sequestering ribosomes; reducing the number of uAUGs or uORFs within the 5′ UTR can alleviate this repression, enhancing the overall translation of the CDS [61]. Furthermore, the Kozak sequence, a crucial nucleotide sequence located immediately downstream of the eukaryotic mRNA 5′ cap structure, typically exemplified by GCCACCAUGG, facilitates the initiation of translation by binding with initiation factors in capped mRNAs, analogous to the Shine–Dalgarno (SD) sequence in prokaryotes [62, 63]. However, UTRs optimization remains largely empirical due to the complex translational regulatory mechanisms involved.

### 3.3 Computational Methods in Sequence Optimization

**FIGURE 4:**
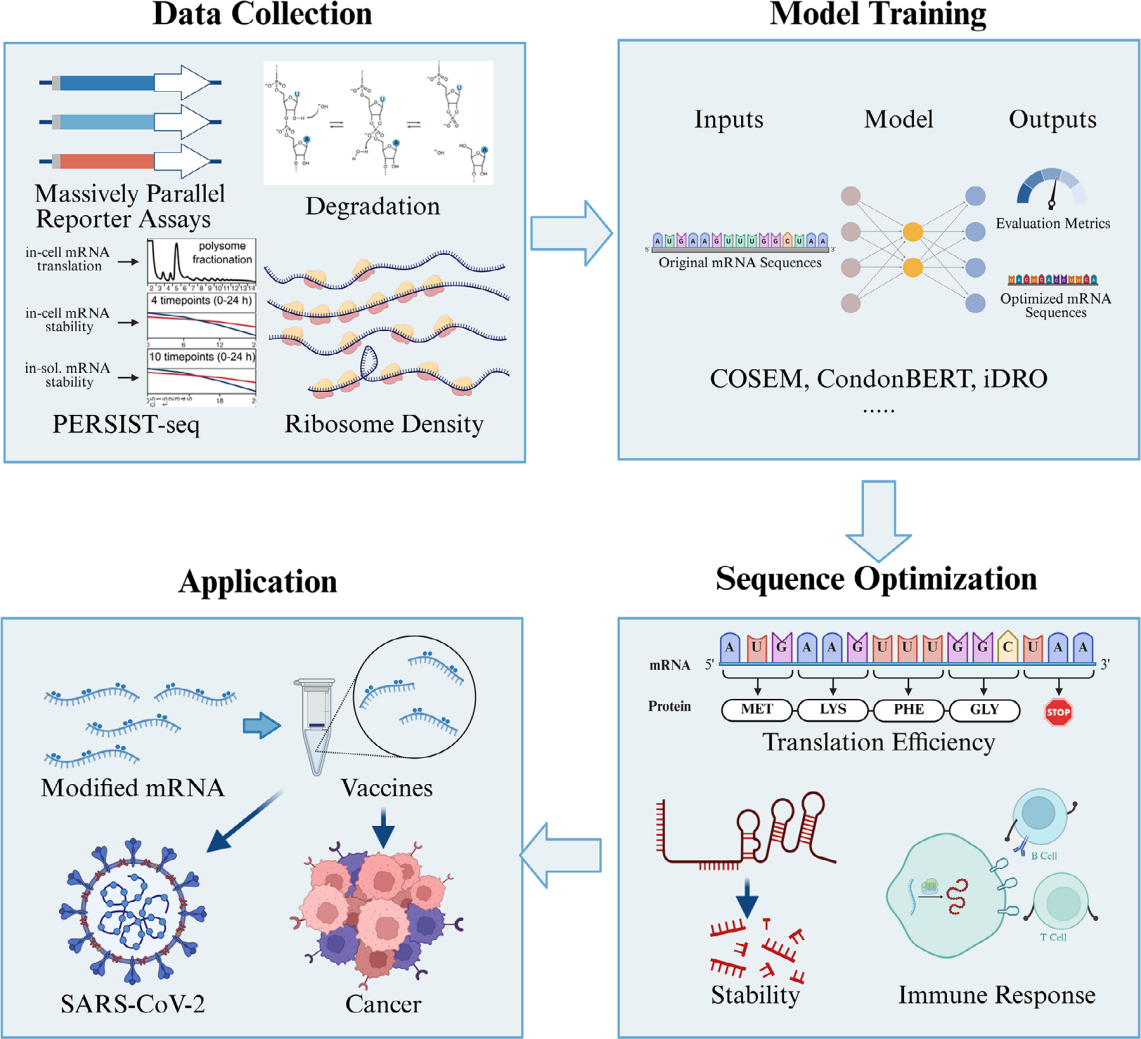
The role of artificial intelligence in mRNA sequence design. This figure demonstrates the application of artificial intelligence in mRNA sequence design. The process begins with acquiring high‐quality, labeled experimental data for effective model training. The trained model is then used to predict the properties of mRNA sequences, and the sequences with better performance are screened out, from which the commonality of their sequences is obtained to guide subsequent modification. After multiple rounds of iterative optimization, the final sequence candidates were gathered and promoted to clinical application. This strategy introduces a novel pattern for mRNA vaccine sequence design, leveraging collective expertise and computational power to optimize sequences. (Created with BioRender.com)

Designing the CDS and UTRs for mRNA vaccines requires careful consideration of multiple factors, including translation initiation, sequence stability, localization, immune response, and so on. The primary challenge lies in the effective balance between these factors, as CDS and UTRs' sequence context is highly specific and can vary significantly. This variability complicates the identification of universally optimal sequences. Data‐driven approaches offer a promising solution to these challenges. By leveraging large datasets and advanced analytical techniques, these methods can uncover complex patterns and relationships within the sequence data. Recent advances in artificial intelligence methods enable us to fully leverage high‐throughput experimental results and comprehensively optimize the aforementioned features from a global optimization perspective (Figure 4). Subsequently, we will introduce the applications of artificial intelligence methods in mRNA sequence optimization.

Regarding CDS design, methods such as the neural network, which predicted ribosome density and simulated translation by incorporating RNA secondary structures, have shown high predictive accuracy in humans and yeast, altering protein yields significantly [64]. Similarly, the Codon‐Specific Elongation Model (COSEM) has provided profound insights into ribosome dynamics, enhancing the accuracy of protein expression predictions and facilitating CDS optimization for *Escherichia coli* and others [65]. For instance, in *Salmonella enterica* serovar Typhimurium, optimization of the manA and ova genes using COSEM‐based algorithms yielded a threefold increase in protein yield compared to wild‐type and commercially optimized sequences. Recently, CodonBERT integrated the ProteinBERT architecture with extensive RNA‐seq data and evaluated it using the CAI and MFE [66]. This provided a customized approach for codon optimization specific to particular organisms, demonstrating its flexibility and accuracy. RNop is a recent DL‐based mRNA optimization method, which designs four loss functions to respectively regulate sequence fidelity, optimize species‐specific codon adaptation, enhance tRNA availability, and improve mRNA secondary structure. Experiments demonstrate that RNop ensures high sequence fidelity, and the optimized mRNA sequences lead to a significant increase in the expression level of functional proteins compared to the control group, with a maximum elevation of 4.6‐fold. It outperforms existing methods in both quantitative metrics and experimental validation [67].

For UTRs, current research combines massively parallel reporter assays (MPRA) with DL networks to identify potential regulatory mechanisms from large‐scale UTR sequence datasets. Seelig et al. conducted experiments on both 5′ UTRs and 3′ UTRs, identifying UTR sequences that potentially enhance translational efficiency [68, 69].

Furthermore, some researchers have attempted to use data‐driven methods to design both CDS and UTR. For instance, the integrated deep‐learning‐based mRNA optimization (iDRO) algorithm optimized mRNA sequences by employing a BiLSTM‐CRF (bidirectional long‐short‐term memory with conditional random field) model for CDS optimization and an RNA‐Bart (bidirectional auto‐regressive transformer) transformer‐based model for generating UTRs, respectively [70]. This integrated approach has demonstrated superior performance in enhancing protein expression. For real‐world applications, iDRO was applied to optimize mRNA sequences for the SARS‐CoV‐2 spike protein, including variants like Alpha, Beta, Gamma, Delta, and Omicron. Structural analyses revealed that iDRO‐generated UTRs had fewer miRNA binding sites and lower MFE than those of Pfizer‐BioNTech's BNT162b2 and Moderna's mRNA‐1273 vaccines [70, 71, 72]. Notably, this method considered the UTR and CDS regions separately. Nevertheless, optimizing each element at once remains challenging due to the limitations of our current data and algorithms.

A promising approach is the dual crowdsourcing strategy, whose emergence has provided new possibilities for RNA sequence design and the application of data‐driven artificial intelligence methods. A prominent example involves utilizing the Eterna RNA design platform to generate short RNA sequences with specific characteristics [73], followed by launching the OpenVaccine competition on Kaggle to predict RNA degradation under experimental conditions through machine learning (ML). Several high‐performing teams have successfully improved upon the benchmark DegScore regression model [74, 75]. Furthermore, this innovative strategy enables direct mRNA design. PERSIST‐seq, a high‐throughput platform capable of evaluating mRNA translation efficiency, intracellular stability, and solution stability, leverages collaborative efforts from the Eterna community to collect extensive mRNA designs, effectively addressing the limitations of traditional methods that focus solely on single aspects of mRNA design [13]. This collaborative and multifaceted approach can significantly advance RNA sequence design. In Table 1, we present a series of recent computational studies for reference.

**TABLE 1:** mRNA vaccine sequence optimization computational methods.

Application	Model	Practical advantages	Practical limitation	Year
UTR design	Optimus 5‐Prime [76]	Strong generalization from being trained on large‐scale experimental data (MRL). Allows for tunable protein expression to achieve specific, targeted output levels.	Further algorithmic optimizations are needed to avoid local optima. Performance is not uniform across all cell types.	2022
	3UTRBERT [77]	Identifies key regulatory elements in the 3′ UTR, such as RBP binding sites, to guide design. Interpretable model helps researchers understand the rationale behind its predictions	The model was pretrained solely on human 3′ UTRs, without accounting for the complexities introduced by the CDS region. The lack of sufficient functional data.	2024
	Smart5UTR [78]	Specialized for m1Ψ‐modified mRNA, which is common in modern therapeutics. Strong in vivo validation in mice showed significantly enhanced antibody titers for mRNA vaccines.	Trained on a single cell line (HEK293T), creating a high risk of poor performance in other therapeutically relevant tissues.	2024
CDS design	Robert Tunney et al. [64]	Accurately predicts ribosome density along an mRNA, which is useful for mechanistic understanding of translation bottlenecks.	The model was primarily trained and tested on yeast mRNA data, and its generalization ability in other biological systems requires further validation.	2018
	COSEM [65]	Provides a detailed, mechanistic simulation of the entire translation process for precise predictions.	The COSEM model relied on multiple parameters, which must be estimated and inferred based on the specific organism and growth conditions.	2019
	Wayment‐Steele et al. [59]	Directly optimizes for mRNA structural stability, which can increase half‐life by at least twofold and help address cold‐chain storage issues.	Based on a simplified model of RNA degradation that may not capture all in vivo enzymatic pathways. Lacks extensive in vivo validation demonstrating a corresponding increase in functional protein output.	2021
	ICOR [79]	ICOR utilized a bidirectional long short‐term memory (BiLSTM) architecture, allowing it to capture the sequence and contextual information of codon usage. ICOR was evaluated on multiple metrics, demonstrating comprehensive optimization performance.	The ICOR model was based on data from the *Escherichia coli* genome, and its effectiveness in other hosts had not yet been validated.	2021
	LinearDesign [13]	Exceptionally fast, enabling rapid design‐test cycles for many candidates. The algorithm could simultaneously optimize mRNA secondary structure stability and codon usage.	Does not explicitly model or optimize for immunogenicity, which must be assessed separately.	2023
	CodonBERT [66]	Flexible BERT‐based architecture effectively captures long‐range codon dependencies.	The method of filtering tissue‐specific data using TPM values was suboptimal, as the results show overly similar codon preferences across different tissues, compromising the model's specificity.	2024
	DERNA [80]	DERNA could simultaneously consider MFE and CAI, resulting in a more balanced RNA sequence. By utilizing a dynamic programming algorithm, DERNA solved the problem within reasonable time and space complexity.	The outcome is highly sensitive to a user‐defined weighting parameter, which may require iterative testing.	2024
	RNop [67]	Comprehensive multi‐objective optimization of four key metrics: fidelity, codon adaptation (CAI), tRNA availability (tAI), and structure (MFE). High‐speed and high‐fidelity design avoids unintended mutations, a risk in some probabilistic models.	“Black box”like deep learning model makes the design principles it learns difficult to interpret.	2024
UTR and CDS design	iDRO [70]	First algorithm to optimize the full mRNA transcript (5′ UTR, CDS, and 3′ UTR) in an integrated fashion.	Computationally expensive and still struggles to fully model long‐range interactions across the entire transcript. Lacks critical in vivo validation in animal models; validation was performed in vitro only.	2023
mRNA design	RNAid [81]	A flexible, open‐source platform that allows researchers to define and customize their own optimization constraints and objectives.	mRNAid only optimized the local MFE at the 5′ end. Calculating the global MFE is highly time‐consuming, and optimizing the MFE of the entire transcript may require substantial computational resources	2022
	Leppek et al. [12]	PERSIST‐seq allowed for the simultaneous screening and analysis of hundreds of mRNA sequences, significantly improving research efficiency and data throughput. The Eterna platform engaged citizen scientists to design highly diverse mRNA sequences, enhancing the diversity and potential of sequence design.	An experimental method that is technically complex and expensive, making it inaccessible to many labs. Results are primarily from a single cell line (HEK293T) and lack in vivo validation in animal models.	2022

## 4 mRNA Modification

mRNA modifications involve chemically altered nucleotides that are widespread in natural mRNAs and alter the chemical and physical properties of mRNA molecules. Extensive research over the past decade has demonstrated that nucleotide modification strategies can reduce immunogenicity while enhancing mRNA stability [10, 82]. This section introduces common modification, existing detection methods for these modifications, and computational methods in predicting mRNA modification sites. Additionally, we explore the utility of modification strategies in mRNA vaccine development.

### 4.1 Summary of Major Modifications Types on mRNA

In modifying mRNA, N6‐methyladenosine (*m^6^A*) is the most prevalent and abundant in eukaryotes [83]. It is distributed throughout the mRNA sequence but is enriched near stop codons and in the 3′ UTRs, often found in the consensus motif DRACH (R = G or A; H = A, C, or U) [84]. *m^6^A* plays a crucial role in regulating mRNA stability and translation efficiency and is closely linked to cell fate decisions, such as the differentiation and functional regulation of T cells [85]. N1‐methyladenosine (*m^1^A*) modification is closely related to *m^6^A*. They can interconvert under alkaline conditions (Dimroth rearrangement) and share some regulatory factors [86, 87]. *m^1^A* modification generally affects RNA base pairing, thus influencing the structure and function of the target RNA molecule [88]. Additionally, *m^1^A* may regulate translation initiation by altering secondary/tertiary structures or recognizing translation initiation sites (TISs), thereby promoting translation [89]. Adenosine‐to‐inosine (A‐to‐I) editing is another widespread co‐transcriptional and posttranscriptional modification in mammals [90]. While A‐to‐I editing occurs less frequently in coding regions, it influences protein translation and function by altering codons [91, 92]. In UTRs, A‐to‐I RNA editing can regulate RNA transport, translation, and degradation [93]. This editing mechanism and *m6A* and *m1A* modifications underscore the complex regulation of mRNA stability, translation, and overall gene expression.

Pseudouridine (*Ψ*) is the C5‐glycoside isomer of uridine, characterized by the connection of the C5 atom (instead of N1) to the C1′ atom of the ribose. This structural feature is found in almost all types of RNA, including coding and noncoding RNA, and is highly conserved across different species [94]. Ψ regulates pre‐mRNA processing, mRNA structure, stability, translation accuracy, and termination [95, 96, 97]. N1‐Methylpseudouridine (*m^1^Ψ*) is a derivative of Ψ, where the N1H position is methylated, catalyzed by pseudouridine methyltransferase (Nep1) [98]. The N1 position of Ψ contains an additional hydrogen bond donor group (*N^1^H*), providing extra basic properties. This modification significantly reduces mRNA immunogenicity and can enhance translation efficiency in certain contexts [99]. 2‐Thiouridine (*s^2^U*) is another modification that may reduce the activity of RIG‐I, thereby mitigating the immune response to mRNA [100]. These modifications play a crucial role in reducing the innate immune detection of mRNA, allowing for more stable expression of modified transcripts.

5‐Methylcytidine (*m^5^C*), occurring at position 5 of cytidine residues in both DNA and RNA, is a prevalent epitranscriptomic mark stabilizing RNA structures and modulating their functions across various types of RNA [101, 102, 103]. Binding proteins, such as Aly/REF export factor (ALYREF) and Y‐box binding protein 1 (YBX1), recognize m5C to influence RNA stability, nuclear export, and translation, making m5C a potential therapeutic target for diseases such as cancer [104, 105]. N4‐acetylcytidine (*ac^4^C*) is the only described acetylation event in eukaryotic RNA [106]. In mRNA, ac4C significantly stabilizes and promotes protein translation in the coding sequence, possibly affecting interactions with corresponding tRNAs during translation [107]. In the 5′ UTR, *ac^4^C* affects translation initiation in a position‐specific manner: *ac^4^C* modifications adjacent to strong AUG start codons can inhibit translation, while those downstream of weak translation start sites can enhance translation [108].

N7‐methylguanosine (*m^7^G*) modulates mRNA transcription, enhancing transcription efficiency and influencing cell proliferation and tumorigenesis [109]. Present in approximately 0.4% of guanosine residues, a level comparable to that of *m^1^A*, *m^7^G* caps are recognized by eIF4E and the cap‐binding complex (CBC) composed of CBP80 and CBP20 [110, 111, 112]. This recognition affects RNA maturation, nuclear export, and translation. Regarding internal *m^7^G* modification, recent studies have revealed that the IGF2BP protein family, particularly IGF2BP3, could preferentially bind to internal *m^7^G* on mRNA and promote the degradation of these *m^7^G*‐modified transcripts [113]. The *m^7^G* cap regulates multiple stages of mRNA, including pre‐mRNA splicing, nuclear export, transcription elongation, translation, and degradation, thereby indirectly increasing ribosome synthesis and translation rates [114]. Besides, for the four types of ribonucleotides, the 2'‐O‐methylation (*2'‐O‐Me*) modification can shield RNA molecules from degradation by nucleases, enhance their hydrophobic properties, and influence their interactions with proteins and other RNAs [115].

### 4.2 Detection Methods

Historically, RNA modifications were identified using one‐dimensional and two‐dimensional thin‐layer chromatography [116]. This technique exploited the differences in the chemical and physical properties between modified and unmodified nucleotides. Recently, this method has been utilized in the SCARLET detection technique, which was developed to assess *m^6^A* at single‐nucleotide resolution in specific transcripts quantitatively [117]. Another approach involved coupling HPLC with mass spectrometry, allowing for the simultaneous measurement of numerous modifications, albeit with limitations in sensitivity [118]. Next‐generation sequencing (NGS) has revolutionized many fields of biology, including RNA research [119]. All RNA sequencing NGS library generation protocols include a reverse transcription step. This step is sensitive to specific RNA modifications that block or slow down reverse transcriptase or induce nucleotide misincorporation in cDNA. RNA modifications can be identified by aligning the RNA sequence with its genomic counterpart, facilitating the identification of diverse RNA modifications [120]. Additionally, RNA modifications, which are typically invisible to reverse transcriptase, can be selectively altered through chemical treatments, leading to misincorporations or truncations during reverse transcription [121]. This method enabled precise mapping of RNA modification levels at single‐nucleotide resolution but was limited to modifications that affect reverse transcriptase or those sensitive to specific treatments.

Moreover, antibody‐based methods could enrich modified RNA fragments by specifically recognizing modified nucleotides. Initially used for detecting m6A, RNA immunoprecipitation (RIP) techniques have been extended to other modifications such as *m^1^A*, *m^5^C*, and *m^7^G* [89, 122, 123, 124]. While powerful and straightforward, this approach relied on the availability of highly specific antibodies and could not provide single‐nucleotide resolution. The mRNA Interactome Capture and Localization with Individual‐nucleotide Resolution Crosslinking and Immunoprecipitation (mi‐CLIP) technique enhanced the resolution of RIP by cross‐linking modification‐specific antibodies with RNA, generating distinct mutation patterns during reverse transcription [125]. In addition to antibody‐dependent methods, independent techniques, such as *m^6^A*‐REF‐seq (*m^6^A*‐sensitive RNA‐Endoribonuclease‐Facilitated sequencing) and DART‐seq (deamination adjacent to RNA modification targets), have been developed for *m^6^A* mapping [126, 127]. While promising, these methods have limitations. For example, *m^6^A*‐REF‐seq can only identify *m^6^A* sites within specific sequence contexts. In contrast, the GLORI (glyoxal and nitrite‐mediated deamination of unmethylated adenosines) method could address the limitations of traditional *m^6^A* detection techniques by significantly enhancing detection accuracy and sensitivity [128, 129, 130]. This method represents a significant advancement, facilitating further studies on the role of RNA modifications in gene expression regulation.

The methodologies employed for detecting and quantifying mRNA modifications each exhibited distinct characteristics, encompassing the range of detectable modification types, measurement precision, sensitivity, and convenience. Consequently, each technique possesses unique strengths and potential applications. For example, antibody‐based methods remain invaluable for rapid and high‐throughput screening scenarios. Furthermore, these varied techniques provide synergistic insights, each offering distinct detection preferences, context‐specific information, or the ability to capture dynamic modifications, thereby establishing a solid foundation for further elucidating the role of modifications in mRNA expression.

### 4.3 Computational Methods in mRNA Modification

**FIGURE 5:**
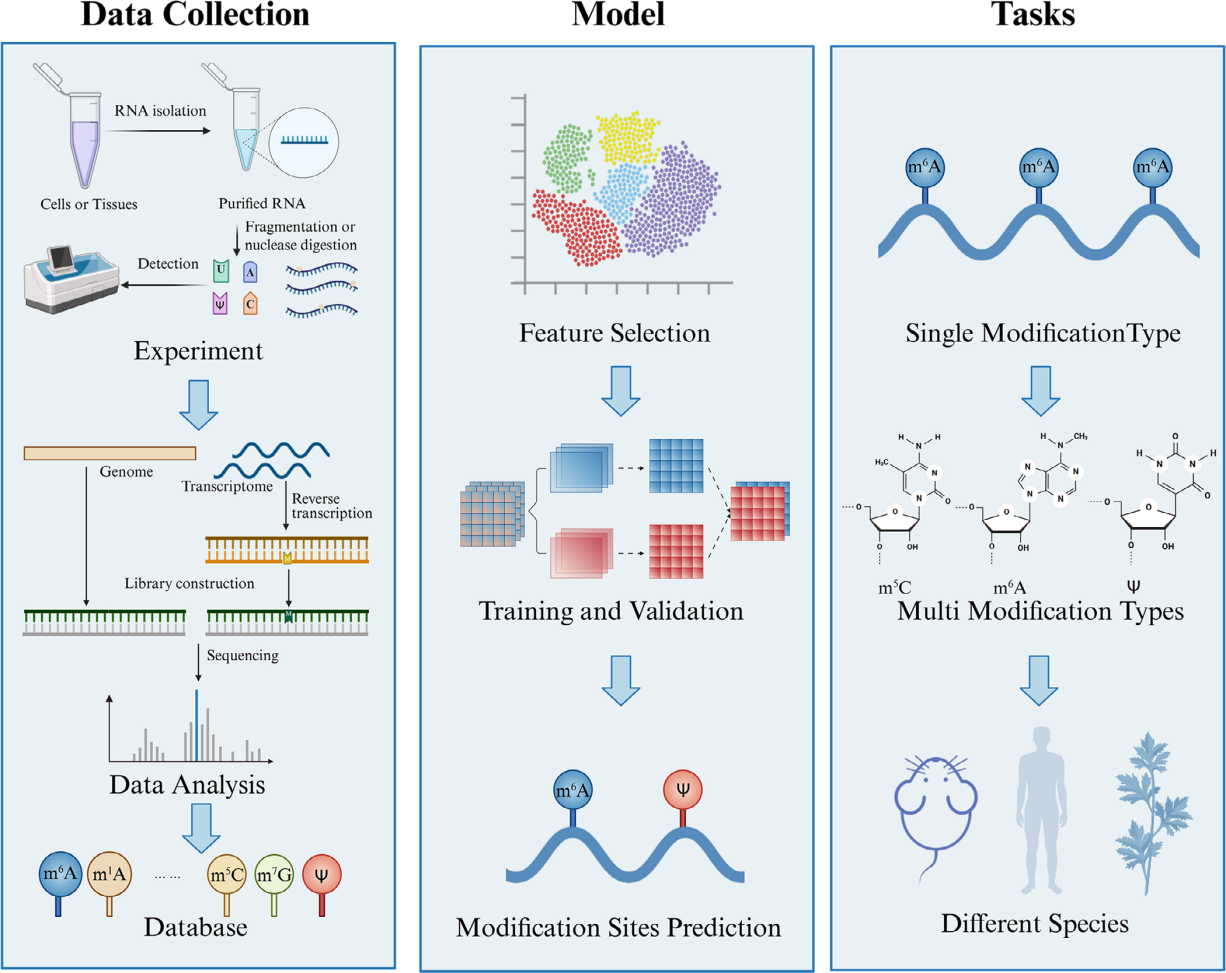
The application of computational methods in RNA modification site prediction. This process is divided into three main stages: data collection and processing, model training, and modification site prediction. Common experimental methods, such as SCARLET, DART‐seq, and GLORI, can be used for detecting RNA modifications. Currently, widely used databases containing mRNA modification data are GEO, MODOMICS, RMBase, and others. The models can be categorized into traditional machine learning and more recent deep learning models. Depending on the data and models used, predictions can be made for single types of modification sites, multiple types of modification sites, and cross‐species modification sites. (Created with BioRender.com)

While biochemical and high‐throughput sequencing methods for identifying RNA modification sites have yielded effective results, they are typically time‐consuming and labor‐intensive. With the increased sequencing data generated, computational algorithms have been employed to predict RNA modification sites (Figure 5). Currently, most benchmark datasets in this field are sourced from the Gene Expression Omnibus (GEO) [131]. Several specialized databases, such as MODOMICS, have also been developed to catalog posttranscriptional modifications [132]. RMBase is a widely utilized RNA modification database, and its latest version, RMBase v3.0, serves as a comprehensive platform for the integrated analysis of RNA modification profiles, mechanisms, interactions, and functions across various species, supported by extensive datasets and innovative analytical tools [133]. Studies on RNA modifications utilize various features for ML, including *K*‐nucleotide frequencies (KNF), position‐specific dinucleotide sequence profile (PSDSP), *k*‐spaced nucleic acid pairs (CKSNAP), and so on [134, 135]. They also incorporate binary variables, evolutionary conservation scores, RNA structure predictions, RNA binding protein annotations, and gene characteristics [136]. Various RNA modifications have been extensively studied and predicted using ML and DL approaches [136].

With the advancement of high‐throughput sequencing technologies, substantial RNA modification data have been collected. However, most existing methods struggle to distinguish between different types of modifications on the same RNA sequence. For example, adenosine can undergo m1A, m6A, and A‐to‐I modifications, which are challenging to identify concurrently. Computational platforms or methods such as iRNA‐PseColl, iMRM, and DeepMRMP (Multiple Types RNA Modification Sites Predictor) have been developed, each exhibiting varying accuracies [137, 138, 139]. Additionally, RNAmod is an interactive platform designed for analyzing and visualizing mRNA modifications across 21 species [140]. These methods often employ similar techniques and algorithms but demonstrate differing levels of accuracy for different modification types. Besides, recent advancements in artificial intelligence have led to the development of models like MRM‐BERT, TransRNAm, Rm‐LR, and MultiRM [141, 142, 143, 144]. These predictive models use transformer architectures or bilinear attention mechanisms to forecast a range of RNA modifications accurately. For example, Rm‐LR utilized sophisticated strategies such as bilinear attention networks and dual independent pretrained models to enhance pattern recognition and interpretability. Furthermore, MRM‐BERT can make predictions across different species regarding their RNA modifications.

In conclusion, while several strategies have been utilized in the computational prediction of RNA modifications, the accuracy and generalization of these methods could be further enhanced with increased data availability. This underscores the need for more experimental data and the development of algorithms that are better aligned with the prediction of mRNA modification sites to enhance their robustness and applicability. The advancement of these predictive methods is particularly crucial for mRNA vaccine development. Enhanced modification sites prediction capability will ultimately accelerate the optimization of mRNA vaccines by enabling precise control over modification patterns that balance immune activation with mRNA stability.

## 5 mRNA Delivery System Optimization

Efficient delivery systems are essential for mRNA vaccine development. This section presents various delivery platforms, focusing primarily on LNPs. We examine the structure and properties of LNPs, and explore how computational approaches advance their design through high‐throughput screening. This systematic analysis aims to accelerate the optimization of mRNA vaccine delivery systems.

### 5.1 Summary of mRNA Delivery Systems

**FIGURE 6:**
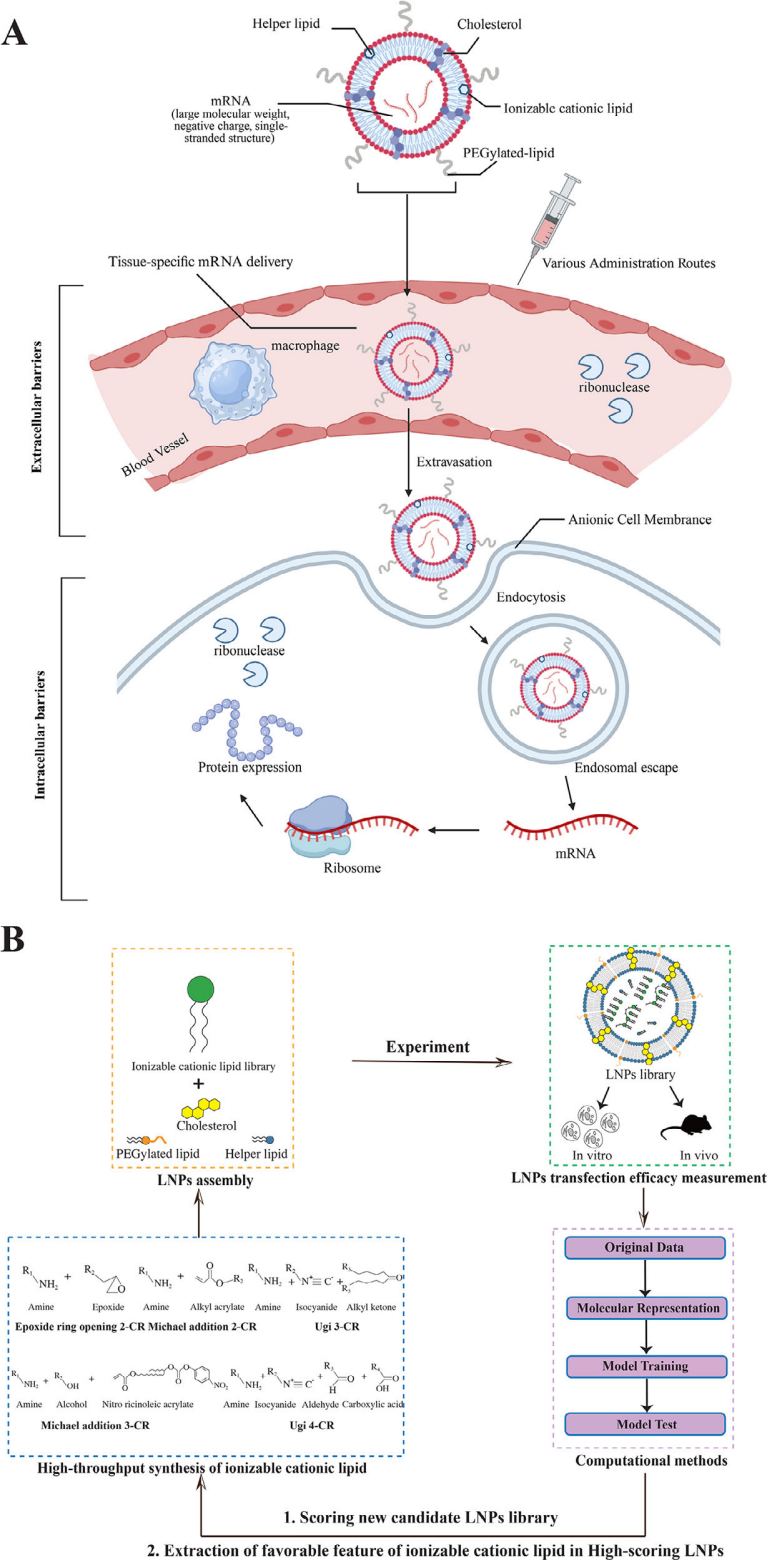
Mechanism of LNP‐facilitated mRNA delivery and computational high‐throughput screening of LNPs. (A) The mechanism through LNPs facilitates the transport of mRNA across extracellular and intracellular barriers. mRNA is a biomolecule with a large molecular weight, a negative charge, and a single‐stranded structure. After entering the blood vessel through various administration routes, LNPs protect mRNA from degradation by macrophages and ribonucleases, and deliver it to specific target tissues (achieved through selective organ targeting LNPs). Following extravasation, LNPs assist the negatively charged mRNA in entering the cell through the positively charged cell membrane via endocytosis. Subsequently, through endosomal escape, mRNA is released from the endosome into the cytoplasm, enabling its function. (Created with BioRender.com). (B) Computational methods‐based high‐throughput screening of LNPs. First, a library of ionizable cationic lipids is synthesized using combinatorial chemistry for high‐throughput screening (blue), and then integrated with cholesterol, helper lipid, and PEGylated lipid to form an LNP library (yellow). The transfection efficiency of LNPs is measured in vitro (e.g., HeLa cell line) or in vivo (e.g., mouse models) as the original data input for the model (green). After molecular representation of LNPs, the model is trained to predict transfection efficacy (purple). The trained model is then used to score a new candidate library, and the common features of ionizable cationic lipids in high‐scoring LNPs are identified to guide the synthesis of new high‐quality ionizable cationic lipids.

Successful mRNA vaccine delivery requires reaching the cytosol of target cells to initiate translation and induce antigen‐specific immune responses [145]. Naked mRNA faces multiple biological barriers: First, mRNA is a negatively charged biomolecule, which hinders its ability to penetrate the cell membrane. Second, mRNA is highly susceptible to degradation due to its single‐stranded structure and the ribonuclease‐rich environment within the body [3, 146]. Lastly, for mRNA to exert its function, it must escape from endosomes; however, a significant portion of mRNA becomes trapped within endosomes upon cellular entry [147]. Therefore, in order to overcome biological obstacles and attain the best possible mRNA expression, mRNA vaccines rely heavily on the development and use of sophisticated delivery vectors [148] (Figure 6A).

A diverse array of delivery vectors has been developed, which can be classified into naturally derived nanoparticles, inorganic delivery systems, and organic vectors [149]. Despite their varied origins and distinct features, these delivery vectors have exhibited promising efficacy and favorable biosafety profiles in preclinical studies, thus facilitating the clinical application of mRNA vaccines.

Naturally derived nanoparticles primarily encompass engineered virus‐like particles (VLPs), protein/peptide assemblies, extracellular vesicles (EVs), and DNA nanostructures. These naturally derived nanoparticles demonstrate superior transfection efficiency. However, they also present distinct limitations, such as the potential to elicit undesired immune responses and the risk of genomic integration [150]. Zhang et al. developed the selective endogenous encapsidation for cellular delivery (SEND), a system that employs the engineered retrovirus‐like protein PEG10 to encapsulate, secrete, and deliver specific RNA molecules. This innovative approach demonstrates reduced immunogenicity compared to traditional viral vectors [151].

In recent studies, inorganic delivery systems such as gold nanoparticles (AuNP), iron oxide nanoparticles, and quantum dots have been reported as alternative gene delivery vehicles [152]. Inorganic nanoparticles are nontoxic, hydrophilic, biocompatible, and highly stable compared with organic materials [153]. However, they are usually associated with complex synthesis and modification as well as biosafety concerns [154].

Organic vectors include LNPs, polymer‐based nanoparticles, and hybrid nanoparticles [149]. Polymer‐based nanoparticles, primarily including polyethyleneimine, polyester, poly(amino acids), and dendrimers, have emerged as promising candidates in recent years due to their versatile structures, facile functionalization, and robust stability [155, 156]. Hybrid nanoparticles combine the complementary properties of lipid and polymeric nanomaterials [157].

This review emphasizes LNPs as the most extensively validated delivery vectors in current research. Their optimization through artificial intelligence integration represents a significant opportunity for advancing mRNA vaccine development.

### 5.2 Property Landscape of LNPs

#### 5.2.1 Formulation of LNPs

The traditional LNPs consist of an ionizable cationic lipid, cholesterol, a helper lipid, and a PEGylated lipid (polyethylene glycol, PEG). These components aggregate to form a membrane structure that encloses and safeguards the mRNA cargo [158]. Several self‐assembled LNP structures have been described in the literature, including multilamellar vesicles [159], nanostructured core particles [160], and homogeneous core–shell structures [161]. Each lipid component serves a distinct function in cargo encapsulation and delivery efficacy [162] (Figure 6B).

Ionizable cationic lipids serve as an essential component in LNP formulations, with their acid dissociation constant (pKa) determining the ionization behavior and surface charge of LNPs, consequently influencing their stability and toxicity [163]. They play a crucial role in promoting membrane fusion/disruption, endosomal escape, and subsequent cargo release into the cytoplasm [164]. The chemical structure of ionizable cationic lipids comprises three main components: the amino head group, the hydrophobic tail, and the internal linker [160]. Depending on the number of amino groups, ionizable cationic lipids can be classified as either single‐amino or multiple‐amino lipids. DLin‐MC3‐DMA (MC3), SM‐102, and ALC‐0315 are the most extensively studied single‐amino lipids. These compounds represent the only ionizable cationic lipids that have received FDA approval for RNA delivery [30, 31, 32]. As for the multiple‐amino lipids, Song et al. developed novel lipids 4N4T with a positively charged core formed of four tertiary amine nitrogen atoms, which had higher delivery efficiency and triggered a more robust and durable immune response than SM102.

Cholesterol enhances the stability of LNPs by filling gaps between lipids and facilitating membrane fusion [165, 166]. Recent studies have elucidated that cholesterol metabolism significantly influences immune cell activities, encompassing activation, differentiation, and functional responses [167]. Phospholipids act as helper lipids that facilitate the formation of LNPs, confer bilayer structural stability, modulate the fluidity of LNPs, and enhance endosomal membrane fusion [168]. Furthermore, phospholipids contribute to the reduction of LNP‐associated toxicity [169]. PEGylated lipids can regulate the size and colloidal stability of LNPs, reduce the aggregation of nanoparticles, and prolong circulation time when administered intravenously in vivo [170, 171].

#### 5.2.2 Characteristics of LNPs

The characteristics of LNPs commonly involve pharmaceutical and biological properties. The pharmaceutical properties include size, zeta potential, stability, storage conditions, and encapsulation efficiency, while the biological properties include targeting ability, endosomal escape, transfection, and immunogenicity [169, 172]. These properties determine whether LNPs can be widely applied. To advance the clinical application of LNPs, making these characteristics more suitable for delivery is necessary.

Optimization strategies for LNPs can be categorized into four main groups: (1) innovative lipid molecule design and screening, (2) formulation adjustment of LNPs, (3) surface modification of LNPs, and (4) choice of administration routes [173]. Su et al. have discovered that certain inherent components of LNPs, such as cholesterol and phospholipid, are not indispensable for delivery and instead lead to inevitable liver accumulation. They have developed a three‐component technology composed of nAcx‐Cm lipids, permanently cationic lipids, and PEG‐lipid, demonstrating that it outperforms in both efficacy and veritable targeting properties for lung delivery while maintaining remarkable stability [174]. Liu et al. developed hundreds of ionizable phospholipids termed iPhos to overcome the limitations of traditional phospholipids, such as structural inflexibility and hard‐to‐access reactions [175]. Intravenously (IV) administered LNPs typically accumulate in the liver and are internalized by liver hepatocytes, thereby greatly limiting the scope of their therapeutic application. Dilliard et al. found that augmenting conventional four‐component LNPs for mRNA delivery to the liver with a fifth component (termed a SORT molecule) can alter the LNPs’ in vivo organ‐targeting properties and lead to the extrahepatic delivery of mRNA [176].

### 5.3 Design and Screening of LNPs

The design and screening of novel lipid molecules has evolved into a high‐throughput approach. This technological advancement enables the generation of large‐scale datasets, facilitating systematic screening processes. Unlike traditional labor‐intensive and costly trial‐and‐error strategies, these approaches integrate computational algorithms to accelerate optimization.

#### 5.3.1 High‐Throughput Synthesis of LNPs

Ionizable cationic lipids have emerged as the main emphasis in the classical formulation of LNPs due to their extensive structural design range, wherein small changes in their chemistry can significantly affect their biological activity [173, 177]. To identify effective ionizable cationic lipids, it is often necessary to compare dozens or even hundreds of chemically diverse candidates. Combinatorial chemistry enables the systematic combination of limited components to generate diverse ionizable cationic lipid variants, establishing comprehensive lipid libraries for screening [178].

The two‐component reaction (2‐CR) was the earliest combinatorial chemistry synthesis, involving direct linkage between lipid tails and amino‐containing headgroups [179]. Two of the most common combinatorial reactions are Michael addition between amines and acrylates or acrylamides and epoxide ring‐opening reactions between amines and epoxides [180, 181, 182]. The three‐component reaction (3‐CR) system was developed to explore a broader chemical space and facilitate the identification of more potent lipids. A Ugi‐based 3‐CR system involving a reaction between an amine, an isocyanide, and a ketone enabled the rapid synthesis of a combinatorial library encompassing 1080 ionizable cationic lipids. This approach led to the discovery of a lipid that activated the STING pathway, thereby enhancing its immunogenicity in the context of an mRNA vaccine [183]. Another 3‐CR system, based on Michael addition reactions, where the nitro ricinoleic acrylate linker was used to connect the lipid tails and headgroups containing primary, secondary, or tertiary amines at its two ends, was employed to generate a library containing over 700 ionizable cationic lipids [184]. Han et al. developed a one‐pot tandem multi‐component reaction (T‐MCR) to enable the fast and facile synthesis of amidine‐incorporated degradable (AID) lipids. The T‐MCR is based on a rationally designed amine‐thiol‐acrylate conjugation reaction, with the reactants being an amine, Traut's reagent (2‐iminothiolane hydrochloride), and alkyl acrylates [185]. Recently, Li et al. introduced a novel high‐throughput synthesis platform based on a four‐component reaction (4‐CR) system, building upon the Ugi‐based 3‐CR system. The ionizable cationic lipid structures are delineated into four discrete elements: an amine head group, a linker, tail 1, and tail 2, corresponding to the reactants in the 4‐CR‐amines, isocyanides, aldehydes, and carboxylic acids, respectively. This platform facilitates systematic design and synthesis of novel ionizable cationic lipids with enhanced efficiency [186].

#### 5.3.2 Computational Methods in High‐Throughput Screening of LNPs

With the rapid advancement of high‐throughput screening technologies, numerous combinatorial lipid libraries have been constructed. Despite this progress, large‐scale experimental screening remains expensive and time‐consuming. Computational methods can play a critical role by performing virtual screening based on existing experimental data, providing optimized directions for subsequent large‐scale experiments, and ultimately reducing experimental costs (Figure 6B).

Applying ML to the screening of ionizable cationic lipids, utilizing the vast amount of existing data, transforms the process from a trial‐and‐error approach to an intelligent, data‐driven strategy. This offers the possibility of quickly screening for effective ionizable cationic lipids that have not yet been experimentally validated [179]. Wang et al. applied LightGBM to the prediction of LNPs' efficacy [187]. In order to forecast binding IgG titer, which indicates the antibody concentration induced by vaccines, researchers gathered data on vaccine‐related information, including antigen protein type and LNP structure, as well as experimental design‐related information such as injection route and subject type, from studies on LNP‐delivered mRNA vaccines. The predictions made by the model were consistent with the findings documented in previous animal studies. While not currently employed for high‐throughput virtual screening of novel LNPs, LightGBM has shown the capability to precisely forecast the results of in vivo experiments involving mRNA‐LNP vaccines. Li et al. attempted ML virtual screening on the Ugi‐based 4‐CR high‐throughput synthesis platform [186]. Using the in vitro transfection efficacy of LNPs evaluated in HeLa cells, the XGBoost model was trained and then used to predict potential high‐transfection‐efficacy lipids in a large library containing many candidate lipids. The screened lipids were not targeted to specific cells but were identified as “promising lipid candidates.” In the following in vivo experiments, LNPs were prepared using either five‐component “selective organ targeting” (SORT) formulations or classic four‐component formulations to verify the transfection efficacy and targeting function of the promising lipid candidates. The experimental results demonstrated that the screened lipid 119‐23 is a potent option for mRNA delivery across a range of cell types in multiple organs [188].

DL offers a promising solution for molecular search space exploration through autonomous feature extraction from raw data, eliminating dependence on manual feature engineering methods. With sufficient high‐quality training data, it can effectively represent the molecular structure and predict molecular properties [189]. Compared to traditional ML methods, DL is capable of learning more comprehensive molecular representations while also offering stronger fitting capabilities [190]. Xu et al. developed the AI‐Guided Ionizable Lipid Engineering (AGILE) platform, a synergistic integration of DL and combinatorial chemistry [191]. AGILE employed a pretrained DL neural network that utilizes a large amount of unlabeled data from combinatorial lipid libraries, adopting a self‐supervised approach to learn differentiable lipid representations. This process used a graph neural network (GNN) to construct a graph encoder. Then, ionizable cationic lipids were synthesized using a Ugi‐based 3‐CR high‐throughput synthesis platform, and LNPs were formulated via a liquid handling robot following a previously established classical four‐component formulation ratio [192]. The model was tailored for transfection potency prediction through supervised fine‐tuning, training the model based on the in vitro screening results of these LNPs in specific target cells, enabling the model to capture the potential transfection ability of molecules. Finally, for a larger candidate library, the trained model was used to predict transfection efficacy in corresponding target cells and select the most promising lipid structures.

The exploration of computational methods to accelerate high‐throughput screening of LNPs is still in its early stages, and related work remains limited. First, LNPs delivery is a complex system. In addition to ionizable cationic lipids, the selection of other components and the optimization of their internal ratios also influence the transfection efficacy of LNPs. Current models capable of virtual screening focus only on classic four‐component formulations or specific five‐component “selective organ targeting” (SORT) formulations, limiting their ability to identify candidate LNPs within these predefined formulations. Consequently, the impact of varying auxiliary lipid components and different component ratios remains unexplored. Second, the characteristics of LNPs extend beyond transfection efficacy. Existing computational methods cannot yet take clinical characteristics, such as the immunogenicity of LNPs, into account. Third, the training data used by computational models are derived from small‐sample in vitro experiments on high‐throughput synthesis platforms. While these data can inform in vivo experiments, a significant gap between in vitro and in vivo results still exists. Due to the complexity of biological systems, such as immune responses, in vitro results often fail to predict in vivo outcomes accurately. The computational models currently employed are relatively simplistic, with limited fitting and generalization capabilities.

Further development of comprehensive models is necessary to expedite the implementation of computational techniques in the virtual screening of LNPs. Moreover, it is essential to investigate models that have improved generalization capabilities in order to consider a broader spectrum of elements that impact the performance of LNPs. It will be crucial to conduct more extensive in vivo studies using actual LNPs and develop measurable metrics for LNPs’ different properties in order to obtain more reliable training data for these models.

## 6 Summary and Prospect

### 6.1 Current Status of mRNA Vaccine Applications in Preclinical and Clinical Settings

Extensive clinical trials are developing mRNA vaccines targeting a broad range of infectious diseases and cancers [193, 194]. These ongoing investigations are systematically evaluating the efficacy and safety profiles of mRNA vaccines for both disease prevention and therapeutic intervention.

The COVID‐19 pandemic provided a decisive proof‐of‐concept: the licensed mRNA vaccines Comirnaty (BNT162b2) and Spikevax (mRNA‐1273) induced potent neutralizing responses and showed favorable real‐world safety [71, 195, 196]. Since then, the infectious‐disease pipeline has expanded dramatically. Approved products now include mNEXSPIKE, a bivalent RBD and NTD COVID‐19 vaccine, and MRESVIA, which targets the pre‐fusion RSV‐F antigen [197, 198, 199]. Late‐stage programs such as the bivalent mRNA‐1083 (COVID‐19 and quadrivalent influenza), the CMV vaccine mRNA‐1647 (glycoprotein B and pentamer), and the trivalent norovirus candidate mRNA‐1403 (VP1 from GII.4, GI.3, GII.3) are all in phase III, while multivalent influenza vaccines (GSK4382276A, mRNA‐1010/1020/1030, MRT5407/5410/5413) and agents for Zika (mRNA‐1893/1325), chikungunya (mRNA‐1388), and Nipah virus (mRNA‐1215) populate phases I–II. Beyond infectious disease, oncology pipelines now test individualized neoantigen therapies (mRNA‐4157, RO7198457, BNT‐122, XH001) that encode up to 34 patient‐specific epitopes, as well as “off‐the‐shelf” constructs targeting viral oncoproteins (BNT‐113 for HPV‐16 E6/E7) or shared tumor antigens (CV‐9202, BNT‐112/116, mRNA‐4106) (Table 2).

**TABLE 2:** Summary of key preclinical animal studies and clinical trials.

Field	Product name	Implication	Mechanism	Phase
Infectious disease	mNEXSPIKE [197]	COVID‐19	encode the receptor‐binding domain (RBD) and N‐terminal domain of SARS‐CoV‐2 spike protein	Approved
	Comirnaty [71]	COVID‐19	encode the SARS‐CoV‐2 spike protein	Approved
	Spikevax [195, 196]	COVID‐19	encode a prefusion‐stabilized SARS‐CoV‐2 S‐protein with the native S1/S2 cleavage site	Approved
	mRNA‐1083 [200]	COVID‐19, Influenza	encode the RBD and N‐terminal domain of SARS‐CoV‐2 spike protein and hemagglutinin (HA) antigens from two influenza A strains (H1N1 and H3N2) and two influenza B strains (B/Victoria and B/Yamagata),	Phase III
	GSK4382276A [201]	Influenza A and B strains	Multivalent seasonal influenza vaccine against four strains	Phase II
	mRNA‐1010 [202, 203]	Influenza A and B strains	encode seasonal HA of Influenza A (H1N1,H3N2) and Influenza B (Yamagata Lineage, Victoria Lineage)	Phases I and II
	mRNA‐1030, mRNA‐1020 [200]	Influenza A and B strains	encode HA and neuraminidase (NA) of Influenza A (H1N1,H3N2) and Influenza B (Yamagata Lineage, Victoria Lineage)	Phase I and II
	MRT5407 MRT5410 MRT5413 [204]	Influenza A and B strains	encode seasonal HA of Influenza A (H1N1,H3N2) and Influenza B (Yamagata Lineage, Victoria Lineage)	Phases I and II
	mRNA‐1018 [201]	Influenza A	encode HA of Influenza A (H5N8,H7N9)	Phase II
	mRNA‐1440 [201, 205]	Influenza A	encode seasonal HA of Influenza A (H10N8)	Phase I
	mRNA‐1851 [205]	Influenza A	encode seasonal HA of Influenza A (H7N9)	Phase I
	MRESVIA [198, 199]	RSV	encode the pre‐fusion form of the membrane‐anchored RSV‐F glycoprotein, derived from an RSV‐A strain	Approved
	mRNA‐1893 [199]	Zika Virus	encode premembrane and envelope E structural proteins (prME)s from the RIO‐U1 Zika virus	Phases I and II
	mRNA‐1325 [206]	Zika Virus	encode the prME from a Micronesia 2007 Zika virus	Phase I
	mRNA‐1647 [206, 207]	Cytomegalovirus Infections	encode 2 CMV antigens including glycoprotein B and the pentameric glycoprotein complex	Phase III
	mRNA‐1403 [208]	Norovirus Infections	encode the major capsid protein (VP1) of three globally prevalent NoV genotypes (GII.4, GI.3, and GII.3)	Phase III
	mRNA‐1405 [209]	Norovirus Infections		Phase II
	mRNA‐1644 [210, 211, 212]	Human Immunodeficiency Virus (HIV)	encode antigen displaying 60 copies of the germline‐targeting engineered gp120 outer domain [eOD‐GT8 60‐mer] and core–based nanoparticle immunogen (Core‐g28v2) of HIV	Phase I
	mRNA‐1574 [201]	HIV	encode HIV trimer	Phase I
	mRNA‐1608 [213]	Herpes simplex virus type 2 (HSV‐2)	encode the HSV‐2 glycoproteins C (gC2), D (gD2) and E (gE2)	Phase II
	mRNA‐1468 [214]	Varicella‐Zoster virus (VZV)	encode the live attenuated VZV (VZV LAV) and adjuvanted VZV gE subunit protein (VZV gE protein/adjuvant)	Phase II
	GSK3903133A [215]	Rabies Virus	encode rabies virus glycoprotein (RABV‐G)	Phase I
	CV7201, CV7202) [216]	Rabies Virus	encode rabies virus glycoprotein (RABV‐G)	Phase I
	mRNA‐1653 [217]	Human metapneumovirus (hMPV) and parainfluenza virus type 3 (PIV3)	encode full‐length membrane‐anchored fusion (F) proteins of hMPV and PIV3	Phase I
	mRNA‐1388 [209, 218]	Chikungunya virus (CHIKV)	encode the full CHIKV structural polyprotein, including capsid and envelope proteins E3, E2, 6k/TF, and E1	Phase I
	mRNA‐1189 [219]	Epstein‐Barr Virus Infection (EBV)	Encode EBV envelope glycoproteins (gp350, gB, gH, gL, and gp42)	Phase II
	mRNA −1215 [220]	Nipah virus (NiV)	encode prefusion stabilized F component covalently linked to G monomer (pre‐F/G) of the NiV‐M strain	Phase I
Cancer	mRNA‐4157 [221, 222]	Non‐Small Cell Lung Cancer (NSCLC), Metastatic melanoma	individualized neoantigen therapy (INT) encoding up to 34 neoantigens inducing specific antitumor T‐cell activation	Phase III
	RO7198457 [223]	Advanced or Metastatic Solid Tumors	INT encoding up to 20 neoantigens inducing specific antitumor T‐cell activation	Phase II
	BNT‐122 [224]	Pancreatic ductal adenocarcinoma (PDAC)	INT encoding up to 20 neoantigens inducing specific antitumor T‐cell activation	Phase II
	XH001 [225]	Advanced gastric, liver and esophageal cancers	INT encoding tumor‐specific neoantigens with potential immunomodulatory and antineoplastic activities	Phase I
	BNT‐113 [194]	Unresectable Recurrent, or Metastatic Head and Neck Squamous Cell Carcinoma (HNSCC)	encode the human papillomavirus type 16 (HPV‐16) oncoproteins E6 and E7	Phases II and III
	mRNA‐4359 [226]	Solid tumors	encode concatemerized program death‐ligand 1 (PD‐L1) and indoleamine 2,3‐dioxygenase 1 (IDO1) antigens	Phase II
	CV‐9202 [227]	NSCLC	encode six non‐small cell lung cancer (NSCLC)‐associated antigens (NY‐ESO‐1, MAGE‐C1, MAGE‐C2, survivin, 5T4, and MUC‐1)	Phase II
	BNT116 [228]	NSCLC	encode six tumor‐associated antigens (TAAs) frequently expressed in NSCLC	Phase I
	mRNA‐4106 [194]	Solid tumors	encode multiple tumor‐associated antigens broadly expressed across a variety of cancers	Phase I
	BNT‐112 [229]	Prostate cancer	Encode the prostate cancer tumor‐associated antigens (TAAs) kallikrein‐2, kallikrein‐3, acid phosphatase prostate, homeobox B13 (HOXB13), and NK3 homeobox 1	Phase I
	mRNA‐4203 [230]	Previously treated, unresectable or metastatic cutaneous melanoma or synovial sarcoma	encode PReferentially expressed Antigen in Melanoma (PRAME)	Preclinical Development

Collectively, evidence from preclinical studies, already‐licensed products, and more than 30 active clinical programs positions mRNA technology as a rapidly deployable, scalable, and immunologically potent platform for both prophylactic and therapeutic vaccination across a broad spectrum of diseases. We present a detailed list of mRNA vaccines currently under investigation, categorized by therapeutic indication in Table 2.

### 6.2 Limitations of Current mRNA Vaccine Technology

Despite the remarkable success and rapid advancement of mRNA vaccine technology, several significant limitations continue to constrain the full realization of mRNA's therapeutic potential. These persistent challenges encompass technical, logistical, ethical, and safety considerations that require comprehensive attention and resolution.

While computational methods demonstrate great promise for mRNA vaccine development, several limitations persist. Due to limited data availability, AI models are susceptible to potential biases and overfitting risks, resulting in weak predictive performance on novel data and potentially compromising generalizability to new scenarios. Given the inherent complexity of biological systems, computational models cannot fully capture their intricate characteristics, leading to discrepancies between computational predictions and experimental validation. Additionally, factors such as delivery efficiency and individual immune variability that are challenging to model accurately create significant gaps between computational optimization and actual in vivo/in vitro efficacy. Future research should prioritize collecting more high‐quality datasets, establishing standardized data processing pipelines, and developing integrated computational‐experimental validation frameworks to bridge the divide between computational predictions and experimental outcomes.

Cold‐chain requirements represent one of the most pressing technical challenges. Most mRNA vaccines necessitate ultra‐low temperature storage conditions, typically ranging from −70°C to −20°C, creating substantial barriers for global distribution, especially in resource‐limited settings and remote areas. This requirement demands sophisticated cold‐chain infrastructure, specialized transportation systems, and trained personnel, significantly increasing costs and limiting accessibility in developing regions where vaccine equity remains critically important.

Rare adverse reactions, though infrequent, pose important safety concerns that warrant continued surveillance and investigation. Reports of myocarditis following mRNA vaccination have raised questions about long‐term safety profiles. Additionally, anaphylactic reactions, though extremely rare, require immediate medical intervention and comprehensive risk assessment protocols [231, 232]. The novelty of mRNA vaccine technology necessitates extended post‐market surveillance to fully understand potential long‐term effects and rare complications.

In addition to technical issues, ethical considerations and privacy concerns emerge from multiple dimensions of mRNA vaccine development and deployment, creating complex challenges that extend beyond traditional biomedical ethics. Issues surrounding equitable global access, informed consent processes, and the prioritization of vulnerable populations during distribution create fundamental moral challenges. The rapid development timeline, while beneficial for pandemic response, has also raised questions about the adequacy of safety data and the ethical implications of emergency use authorizations. Beyond these foundational ethical issues, data privacy concerns have become increasingly prominent as digital health passports and vaccination tracking systems proliferate. The integration of mRNA vaccination records with digital platforms raises questions about personal health data protection, governmental surveillance capabilities, and individual privacy rights. The potential for discriminatory practices based on vaccination status and the long‐term storage of sensitive medical information present ongoing challenges requiring robust regulatory frameworks that address both traditional bioethical principles and modern digital privacy rights.

Addressing these multifaceted limitations through continued research, policy development, and technological innovation is essential for maximizing the transformative potential of mRNA vaccines. Successful resolution of these challenges will not only enhance the current generation of mRNA vaccines but also pave the way for broader applications in cancer therapy, genetic diseases, and personalized medicine, ultimately establishing mRNA technology as a cornerstone of RNA therapeutic.

### 6.3 Summary and Future Prospects of mRNA Vaccine

The development of mRNA vaccines has shown significant progress over the last three decades, providing prompt and effective remedies for newly evolving infectious diseases. However, three major challenges persist: optimizing mRNA sequences for enhanced stability and expression, selecting effective chemical modifications to improve safety and efficacy, and designing efficient delivery systems for precise mRNA delivery. In this review, we discuss these key challenges in detail and highlight how computational methods, particularly DL and ML, show great promise in advancing mRNA vaccine development through sequence optimization, chemical modification guidance, and targeted delivery system refinement.

The integration of large language models and artificial intelligence represents a transformative opportunity for mRNA vaccine development. Advanced AI systems, such as nucleotide language models and multimodal DL architectures [66, 233], hold tremendous potential for achieving global optimization of mRNA vaccine design. These models can simultaneously optimize multiple parameters, including codon usage, secondary structure, and chemical modifications through end‐to‐end learning approaches. By training on vast datasets encompassing genomics, proteomics, and clinical outcomes, future AI systems may enable the rapid design of highly effective vaccines tailored to specific pathogens or even predict optimal vaccine candidates for emerging threats before experimental validation.

Novel delivery systems present another frontier of innovation, with smart nanoparticles and tissue‐specific targeting becoming increasingly sophisticated, while artificial intelligence methods have further accelerated the development process. Future developments may include stimuli‐responsive LNPs that release mRNA cargo in response to specific cellular environments, organ‐targeted delivery systems that minimize off‐target effects, and novel biomaterials that enhance cellular uptake and immunogenicity. The convergence of nanotechnology with personalized medicine will likely enable precision vaccine delivery based on individual genetic profiles, immune status, and demographic characteristics, potentially revolutionizing vaccination strategies for diverse populations.

Looking forward, the convergence of computational biology, artificial intelligence, and advanced manufacturing will likely establish mRNA vaccines as a cornerstone of precision medicine. The development of automated, AI‐driven vaccine design platforms could enable rapid response to pandemic threats within weeks rather than months, while personalized vaccination strategies may optimize individual immune responses and minimize adverse effects. As our understanding of immunology deepens and computational capabilities continue to advance, mRNA vaccines may evolve into highly sophisticated, programmable therapeutics capable of addressing complex medical challenges across multiple disciplines, fundamentally transforming preventive and therapeutic medicine in the coming decades.

## Author Contributions

H.W., R.G., and D.S. contributed to the manuscript writing, while D.L. and X.X. conducted data collection and material organization. Y.L. assisted with manuscript revision. All authors have read and approved the final manuscript.

## Funding

The study was supported by the National Key Research and Development Program of China (No. 2023YFC3403200), the Beijing Nova Program (No. 2023111), the Shanghai Specialized Program Promoting Industrial Development (No. 2023‐GZL‐RGZN‐01005), and the Shanghai Action Plan for Science, Technology and Innovation (No. 24JS2820200).

## Ethics Statement

The authors have nothing to report.

## Conflicts of Interest

Authors Ruichu Gu, Duanmiao Si, Yongge Li, and Han Wen are employees of DP Technology Company. The other authors declare no conflicts of interest.

## Data Availability

The authors have nothing to report.
